# Engagement of patients in religious and spiritual practices: Confirmatory results with the SpREUK-P 1.1 questionnaire as a tool of quality of life research

**DOI:** 10.1186/1477-7525-3-53

**Published:** 2005-09-06

**Authors:** Arndt Büssing, Peter F Matthiessen, Thomas Ostermann

**Affiliations:** 1 Department of Medical Theory and Complementary Medicine, University Witten/Herdecke, Gerhard-Kienle-Weg 4, 58313 Herdecke, Germany; 2 Krebsforschung Herdecke, Department of Applied Immunology, Heinrichstraße 67, 44805 Bochum, Germany

**Keywords:** Questionnaires, Religion and Medicine, Spirituality and Religion, religious practices, coping, chronic disease, cancer, multiple sclerosis

## Abstract

**Background:**

Quality of life is a multidimensional construct composed of functional, physical, emotional, social and spiritual well-being. In order to examine how patients with severe diseases view the impact of spirituality and religiosity on their health and how they cope with illness, we have developed the SpREUK questionnaire. We deliberately avoided the intermingling of attitudes, convictions and practices, and thus addressed the distinct forms and frequencies of spiritual/religious practices in an additional manual, the SpREUK-P questionnaire.

**Methods:**

The SpREUK-P was designed to differentiate spiritual, religious, existentialistic and philosophical practices. It was tested in a sample of 354 German subjects (71% women; 49.0 ± 12.5 years). Half of them were healthy controls, while among the patients cancer was diagnosed in 54%, multiple sclerosis in 22%, and other chronic diseases in 23%. Reliability and factor analysis of the inventory were performed according to the standard procedures.

**Results:**

We confirmed the structure and consistency of the previously described 18-item SpREUK-P manual and improved the quality of the current construct by adding several new items. The new 25-item SpREUK-P 1.1 (Cronbach's alpha = 0.8517) has the following scales: (1) conventional religious practice (CRP), (2) existentialistic practice (ExP), (3) unconventional spiritual practice (USP), (4) nature/environment-oriented practice (NoP), and (5) humanistic practice (HuP). Among the tested individuals, the highest engagement scores were found for HuP and NoP, while the lowest were found for the USP. Women had significantly higher scores for ExP than male patients. With respect to age, the engagement in CRP increases with increasing age, while the engagement in a HuP decreased. Individuals with a Christian orientation and with a religious and spiritual attitude had the highest engagement scores for CRP, while the engagement in an USP was high with respect to a spiritual attitude. Variance analyses confirmed that the SpR attitude and religious affiliation are the main relevant covariates for CRP and ExP, while for the USP the SpR attitude and the educational level are of significance, but not religious affiliation. Patients with multiple sclerosis overall had the lowest engagement scores for all five forms of SpR practice, while it is remarkable that cancer patients had lower scores for HuP and USP than healthy subjects.

**Conclusion:**

The current re-evaluation of the SpREUK-P questionnaire (Version 1.1) indicates that it is a reliable, valid measure of five distinct forms of spiritual, religious and philosophical practice that may be especially useful for assessing the role of spirituality and religiosity in health related research. An advantage of our instruments is the clear-cut differentiation between convictions and attitudes on the one hand, and the expression of these attitudes in a concrete engagement on the other hand.

## Background

Quality of life is a multidimensional construct composed of functional, physical, emotional, social and (newly introduced) spiritual well-being [1,2]. In breast cancer patients, Levine and Targ [3] found significant correlations of spirituality and spiritual well-being with functional well-being, while items pertaining to meaning and peace tended to correlate significantly with physical well-being. However, "spiritual well-being" itself is also a multidimensional construct. Murray *et al*. [4] described the signs of spiritual well-being as "inner peace and harmony; having hope, goals and ambitions; social life and place in community retained; feeling of uniqueness and individuality; dignity; feeling valued; coping with and sharing emotions; ability to communicate with truth and honesty; being able to practice religion; finding meaning". Thus, it is obvious that spiritual well-being is inextricably intertwined with the physical, social and emotional needs of patients.

Within the last years scientific research approved several connections between religion, spirituality, and health. Several studies indicate that religious involvement and spirituality are associated with better recovery from illness, greater longevity, coping skills, health-related quality of life, less anxiety and less depression (reviewed by [5-11]). As mentioned by Koenig [12], the findings are particularly strong in patients with severe or chronic diseases who have stressful psychological and social changes, as well as existential struggles related to meaning and purpose. Surveys suggest that most patients regard their spiritual health and physical health as equally important [5].

However, the measurability of a religious and/or spiritual attitude respectively towards engagement in distinct forms of a spiritual and religious practice is somewhat difficult. Even though the constructs *Religiosity *and *Spirituality *may not be identical, they were interchangeable to a certain degree in their origins. Nowadays, it is well established practice to divide *Religiosity *into the three sub-constructs intrinsic, extrinsic, and quest religiosity [13-16]. In contrast, the construct *Spirituality *is commonly divided into the following sub-constructs: Cognitive Orientation Towards Spirituality, Experiential/Phenomenological Dimension of Spirituality, Existential Well-Being, Paranormal Beliefs, and Religiousness [17].

Due to their close contextual and cultural coherence, several inventories designed to measure spirituality ask for specific and locally valid religious beliefs and practices (i.e. church attendance and praying) and/or assume a belief in God [17-23]. But this may be inappropriate for patients with different religious, cultural or philosophical backgrounds, or atheist or agnostic patients who may still be spiritually oriented, but are not addressed with regard to their distinct forms of practice in the inventories. A useful tool which does not assume a specific belief in God is the *Functional Assessment of Chronic Illness Therapy – Spiritual Well-Being *(FACIT-Sp) [1,2]; however, it differentiates only two factors, i.e. "faith", and "meaning and peace".

Another new inventory is our SpREUK questionnaire, which asks for basic SpR attitudes and convictions (i.e. Search for meaningful support; Positive interpretation of disease; Trust in external guidance; Support in relations with the External through SpR; Stabilization of the inner condition through SpR). In order to examine how patients with severe diseases view the impact of spirituality and religiosity (SpR) on their health and how they cope with illness, we previously performed several studies with this new inventory [24-29]. We found that patients with both a religious and spiritual attitude had significantly higher values in the sub-scales dealing with the search for meaningful support, and the stabilizing effects of SpR than patients without such attitudes, while patients with a non-spiritual religious attitude had lower perception of the beneficial effects of their SpR and had significantly lower scores in the search for meaningful support sub-scale [24-26]. However, no significant differences were found in the SpR attitude groups with regard to the meaning of disease. Reflecting on meaning and sense of life and positive interpretation of disease obviously can have an impact on how patients change their further life, and thus on distinct forms and engagement frequencies of their SpR practice. Depending on the strength of SpR beliefs and convictions some may feel love and concern for others or have a sense of connectedness, but those who are religious or spiritual only literally might not show any concern for others and are self-centered.

Following these results, it is important to analyze the forms of religious or spiritual involvement of the patients, and to connect their engagement with the SpR attitudes and their convictions how this may have an effect on the course of disease. As the original SpREUK does neither ask for distinct forms nor frequency of SpR practices, these topics are addressed in the newly developped SpEUK-P manual [26,27]. To account for the fact of an institutional religion declines in Europe [30], and the alternative use of various existing esoteric and religious resources, we intended to ask for both, the conventional forms of SpR (i.e. praying, service attendance, recitation of distinct texts, reading distinct books, meditation etc.), and a more reflecting or philosophical practice and nature/environment-oriented practice.

In this paper, we aimed to examine the statical properties of this new SpREUK-P module and how it interacts with the given SpREUK-scores.

## Methods

### Procedure and subjects

All individuals were informed of the purpose of the study, were assured of confidentiality, and gave informed consent to participate. The patients were recruited consecutively in the cancer service, the multiple sclerosis service, and two internal medical units of the Communal Hospital in Herdecke (West-Germany). The healthy subjects were recruited among the medical staff of the Community Hospitals in Herdecke and Berlin, staff of an ambulant out-patient care unit in Essen, attendants of a meeting on "Spirituality and Medicine" in Berlin, a *Caritas *congress in Kevealer, and a meeting of contemporary Christian songwriters in Trier. All subjects completed the questionnaires (SpREUK 1.1 and SpREUK-P) by themselves. Demographic information is provided in Table 1.

**Table 1:** Demographic data and SpREUK-P scores of 354 subjects

		**CRP**	**ExP**	**USP**	HuP	**NoP**
	**%**	(45.5 ± 27.6)	(60.9 ± 18.3)	(32.0 ± 24.0)	(74.4 ± 20.0)	(69.9 ± 18.7)
**sex**			*			(*)
female	71	46.6 ± 27.3	62.4 ± 18.1	31.8 ± 24.9	74.7 ± 19.8	71.0 ± 18.3
male	29	42.5 ± 29.0	56.9 ± 18.2	32.1 ± 22.2	73.4 ± 19.9	67.1 ± 19.5

**age**		*			*	
< 30 years	4	**34.0 ± 32.8**	57.4 ± 21.2	**21.4 ± 19.1**	74.5 ± 23.1	61.1 ± 22.9
30–49 years	54	41.5 ± 27.1	60.6 ± 18.3	32.5 ± 24.8	75.8 ± 18.6	69.0 ± 18.1
50–69 years	35	51.8 ± 27.2	62.8 ± 17.7	33.4 ± 23.9	74.8 ± 19.1	71.4 ± 19.7
> 70 years	6	50.7 ± 25.8	55.9 ± 20.4	**25.4 ± 19.1**	60.5 ± 28.4	74.2 ± 14.3

**marital status**		*	**			
married	59	47.8 ± 27.1	58.3 ± 17.5	29.1 ± 21.3	74.0 ± 21.2	68.5 ± 18.6
living with partner	13	**37.6 ± 23.6**	65.6 ± 16.3	32.9 ± 24.9	78.7 ± 16.7	70.4 ± 18.9
divorced	9	48.6 ± 26.8	**72.2 ± 18.1**	**38.9 ± 28.7**	74.1 ± 18.6	78.1 ± 19.7
alone	16	**38.2 ± 30.6**	59.4 ± 22.0	34.1 ± 27.3	73.1 ± 19.3	67.6 ± 19.3
widowed	3	**57.4 ± 30.5**	56.1 ± 11.3	**26.6 ± 21.9**	71.3 ± 19.1	71.5 ± 13.7

**education**^1^		**	**	**	(*)	
level 1	14	44.3 ± 26.1	52.3 ± 18.6	**20.1 ± 19.7**	67.4 ± 22.8	66.4 ± 22.7
level 2	24	**38.2 ± 24.6**	57.5 ± 17.4	**24.3 ± 17.6**	74.1 ± 20.0	70.9 ± 17.7
level 3	40	**52.3 ± 28.8**	63.5 ± 17.5	**38.6 ± 26.4**	76.0 ± 18.8	69.2 ± 18.0
other	13	**36.8 ± 24.8**	67.0 ± 17.6	34.3 ± 21.6	76.5 ± 19.6	75.0 ± 18.1

**disease**		(*)	*	**	**	*
Healthy	50	47.3 ± 28.8	62.3 ± 16.6	36.5 ± 25.3	78.4 ± 15.8	70.5 ± 19.0
Chronic diseases	12	41.6 ± 27.2	64.7 ± 20.9	35,5 ± 26.6	78.4 ± 22.0	73.9 ± 17.3
Multiple Sclerosis	11	**36.2 ± 28.3**	51.9 ± 17.9	**16.2 ± 15.3**	68.3 ± 27.8	60.3 ± 20.7
Cancer	26	46.4 ± 25.5	60.1 ± 19.5	28.6 ± 20.5	68.0 ± 20.4	72.2 ± 16.3

**religious affiliation**		**	*			
Christian	84	50.4 ± 26.2	61.0 ± 17.5	31.8 ± 23.1	74.1 ± 19.9	70.0 ± 19.1
Others^2^	3	**38.3 ± 32.6**	**73.8 ± 18.9**	**46.0 ± 35.7**	72.2 ± 15.5	76.6 ± 13.3
None	13	**16.0 ± 15.4**	55.8 ± 20.8	29.4 ± 27.0	77.1 ± 20.6	67.6 ± 17.5

**spiritual attitude**		**	**	**	*	**
R+S+	47	**57.3 ± 24.3**	66.8 ± 17.0	**42.4 ± 22.2**	76.5 ± 18.2	72.8 ± 16.8
R+S-	25	51.3 ± 25.2	55.3 ± 15.5	**19.6 ± 15.6**	70.5 ± 22.2	67.6 ± 18.0
R-S+	12	**22.9 ± 18.7**	65.5 ± 16.1	**42.2 ± 26.7**	78.0 ± 16.4	75.5 ± 14.7
R-S-	17	**18.8 ± 16.2**	**47.6 ± 18.1**	**12.2 ± 15.3**	71.1 ± 21.8	61.9 ± 19.6

^1 ^Increasing educational level (based on German school system): 1 = secondary education (Hauptschule), 2 = secondary education (junior high; Realschule), 3 = high school education (Gymnasium).

^2 ^Islam, Buddhism, "Christengemeinschaft" and some not-specified confessions. Scores are significantly different (** p < 0.01; * p < 0.05; (*) 0.05 < p < 0.10; ANOVA).

Deviations of >15% from the mean were highlighted.

The sample contained 354 subjects of whom 71% were women. The mean age was 49.0 ± 12.5 years. Half of the subjects were healthy, while among the patients cancer was diagnosed in 54%, multiple sclerosis in 22%, and other chronic diseases in 23% (i.e. Hepatitis C, liver cirrhosis, inflammatory bowel disease, severe hypertension etc.). Patients in final stages of their disease were not enrolled.

### Measures

The SpREUK-P was designed to differentiate spiritual, religious, existentialistic, and philosophical practices. The items were developed with the patients' input (cancer service of the Herdecke Community Hospital) and experts' statements (physicians, therapists, and priest working with patients) [26,27].

According to a previously conducted pilot study on the reliability and factorial validity on the original 18-item SpREUK-P 1.0 version [26], the following scales were derived: (1) conventional religious practice (CRP), (2) nature-oriented practice (NoP), (3) existentialistic practice (ExP), (4) unconventional spiritual practice (USP), and (5) humanistic practice (HuP). As some of the scales had only a few items, eight new questions (No. 19–26) were added, in particular to strengthen the HuP construct. In total, our item pool therefore consisted of 26 items. All items were scored on a 4-point scale (0 – never; 1 – seldom; 2 – often; 3 – regularly). The SpREUK-P scores are referred to a 100% level (4 "regularly " = 100%), which reflects the degree of an engagement in the distinct forms of a SpR practice ("engagement scores").

### Statistical analysis

Reliability and factor analysis of the new inventory were performed according to the standard procedures. In order to eliminate items from the item pool that were not contributing to the questionnaire reliability, the reliability of the scale and distinct sub-scales was evaluated with internal consistency coefficients, which reflect the degree to which all items on a particular scale measure a single (unidimensional) concept. To combine several items with similar content, we relied on the technique of factor analysis, which examines the correlations among a set of variables, in order to achieve a set of more general "factors." VARIMAX-factor analysis was repeated rotating different numbers of items in order to arrive at a convergent solution embodying both the simplest structure and the most coherent.

Differences in the SpREUK scores were tested using ANOVA. We judged p < 0.05 significant, and 0,05 < p < 0.10 as a trend. To test the impact of several variables on the SpREUK sub-scales, we performed analysis of univariate variance (ANOVA).

All statistical analyses were performed with SPSS for Windows 10.0.

## Results

### Reliability

Reliability analysis revealed that item "Church attendance" (P2) had a poor corrected item-total correlation (Table 2) and thus should have been eliminated. However, as this item is of major conceptual importance (also in other questionnaires), we decided not to eliminate it.

**Table 2:** Mean values of the items from SpREUK-P 1.1 and reliability parameters

	**Factors and Items**	Mean value (Score 0–3)	SD	Factor load	Item difficulty	Corrected Item-Total correlation	Alpha if Item deleted (α = 0.8517)
	**Conventional Religious Practice **(α = 0.8642)						
P2	go to church:	1.30	0.94	0.794	0.44	0.116	0.858
P20	participate in religious events:	0.87	0.98	0.779	0.29	0.266	0.851
P19	religious symbols are important in private area	1.37	1.03	0.772	0.46	0.414	0.846
P1	pray:	1.66	0.99	0.692	0.55	0.499	0.843
	**Existentialistic Practice **(α = 0.7797)						
P13	work on my self-realization:	1.65	0.80	0.782	0.55	0.318	0.849
P14	work on my spiritual development:	2.01	0.75	0.667	0.67	0.483	0.844
P11	try to get insight (also into myself):	2.07	0.75	0.606	0.69	0.682	0.838
P15	try to achieve a higher level of consciousness:	1.23	0.91	0.568	0.41	0.531	0.842
P10	reflect upon the meaning of life:	2.08	0.75	0.520	0.70	0.460	0.845
P16	try to convey positive values & convictions to others:	1.91	0.77	0.436	0.64	0.432	0.846
	**Unconventional Spiritual Practice **(α = 0.7535)						
P7	work on a body-mind discipline:	0.92	1.04	0.768	0.31	0.450	0.844
P4	meditate:	0.91	0.93	0.723	0.31	0.471	0.844
P8	perform distinct rituals:	0.99	1.02	0.682	0.33	0.577	0.839
P6	read religious/spiritual books:	1.24	1.15	0.595	0.41	0.371	0.826
P5	recite distinct (i.e. holy) texts:	0.71	0.86	0.534	0.24	0.483	0.844
	**Humanistic Practice **(α = 0.7907)						
P23	try to consider the needs of others:	2.31	0.56	0.774	0.77	0.319	0.849
P22	try to help others:	2.41	0.58	0.746	0.80	0.355	0.848
P24	thoughts are with those in need:	1.98	0.70	0.717	0.66	0.370	0.848
P25	try to do good:	2.30	0.58	0.650	0.77	0.302	0.849
P26	feel connected with others:	2.26	0.66	0.574	0.75	0.340	0.848
P3	try make an effort for other people:	2.21	0.70	0.445	0.74	0.499	0.844
	**Nature-oriented Practice **(α = 0.5853)						
P17	try to be aware of how I treat the world around:	2.42	0.60	0.725	0.80	0.396	0.847
P18	try to have a healing effect on environment:	1.85	0.82	0.616	0.62	0.519	0.843
P9	turn to nature:	2.03	0.81	0.551	0.68	0.244	0.851
	**Guardian Angel**						
P21x	belief in (my) Guardian Angel:	1.79	1.03	0.584	0.60	0.377	0.848

As shown in Table 2, the new construct had a good quality (Cronbach's alpha = 0.8517). The item difficulty (1.65 [mean value]/3) is 0.55. With the exception of item P17 ("I try to be aware of the way I treat the world around me"; item difficulty = 0.81), all values are in the acceptable range from 0.2 to 0.8.

### Factor analysis

Factor analysis revealed a Kaiser-Mayer-Olkin value of 0.79, which as a measures for the degree of common variance, indicates that the item-pool seems to be suitable for a factorial validation. In addition, Barlett's test for non-sphericity was highly significant (p < 0,001).

Primary factor analysis of item pool pointed to a 7-factor solution, which would explain 60.4% of variance. However, due to a low item number in the tentative subscales 6 and 7 (with 2 items each), we favored the more appropriate 6-factor solution, which explains 56.2% of variance and is provided in Table 2.

The 4-item sub-scale CRP had an alpha of 0.8642, the 6-item sub-scale ExP had an alpha of 0.7797, sub-scale USP with its 5 items had an alpha of 0.7535, and the sub-scale HuP with its 6 items had a Cronbach's alpha of 0.7907, while the 3-item sub-scale NoP had an alpha of 0.5853. As the item P21x ("Guardian angel") made up a factor on its own – even in a tentative 5-factor solution -, we decided to use it as a marker item until the construct is revised for this topic. Thus, the internal consistency of the item pool was sufficiently high.

Analysis of the "side-loadings" of the item pool (only values > 0.35 were take into account) revealed that the marker item P21x ("guardian angel") together with items P11 ("get insight") and P10 ("reflect upon the meaning of life") from the ExP sub-scale and item P5 ("recite distinct texts") from the USP sub-scale would load to a tentative sub-scale 7, while item P21x would load only on the NoP sub-scale 5 (0.368). However, this solution would decrease the quality of the other respective sub-scales and thus was rejected. Item P15 ("higher level of consciousness") from the ExP sub-scale also loads on the NoP sub-scale (0.388).

### Relation between SpREUK-P scores and demographic variables

The highest engagement scores were found for HuP and NoP, while the lowest were found for the USP. Means and standard deviations for study variables are provided in Table 1.

Women had significantly higher scores for ExP than male patients, and in trend also for NoP.

With respect to age, the engagement in CRP increases with increasing age, while the engagement in a HuP decreases with increasing age. Although not significant, it is remarkable that the lowest scores for USP were found in subjects < 30 years and > 70 years of age.

With respect to the marriage status, subjects living alone or with a partner but not married had the lowest scores for CRP, whilst widowed patients had the highest engagement scores. This is in agreement with our previous findings that these individuals rely on "external guidance" [25], but not the patients living with an unmarried partner. Divorced subjects had the highest engagement scores for ExP (and, although not significant, for NoP) which is again in accordance with our previous findings that they are highly in search of meaningful support [25].

Engagement in USP, CRP and ExP depended on the educational level: Patients with higher educational level had significantly higher scores than those with a lower level. Only the NoP did not depend on educational level, while for HuP we observed only a trend.

However, patients with MS overall had the lowest engagement scores, while patients with cancer or other chronic diseases did not differ in regard of the engagement in NoP, ExP and CRP. But it is somehow remarkable, that in contrast to patients with other chronic diseases, the cancer patients had lower scores for HuP and USP than healthy subjects.

It is not surprising that individuals without any religious affiliation had the lowest scores for the engagement in CRP, while their engagement in USP was similar to that of Christian subjects. The few individuals with religious affiliation other than Christian had the highest scores for USP and ExP, but due to a too small investigation group, this statement can be valued only as a hint. There were no significant differences between these three groups with respect to an engagement in NoP and HuP.

Since nominational affiliation is not necessarily identical with religiosity or spirituality, we asked whether the patients would describe themselves as religious or spiritual [24-29]. 47 % of the 354 subjects analyzed herein reported themselves as both religious and spiritual **(R+S+)**; 25% as religious, but not spiritual **(R+S-)**; 17% as neither religious nor spiritual **(R-S-)**, while 12% claimed that they were spiritual, but not religious **(R-S+)**. Thus, the numbers of patients with denominational affiliation and self-reported spiritual/religious attitudes are somewhat similar.

However, R+S+ subjects had the highest engagement score for CRP (even higher than that of R+S-). The lower score of subjects with a R-S+ attitude was comparable with that of R-S-. The engagement in an USP was high with respect to a spiritual attitude (R+S+ and R-S+), while the lowest scores were found for R-S- and R+S- subjects. It is remarkable that R-S- subjects had the lowest engagement scores for all five forms of a SpR practice.

At present the item P21x ("belief in (my) Guardian Angel"), which was a factor on its own, should be regarded just as a marker item. We found significant differences in this item between the SpR attitude groups (F = 7.649; p < 0.0001). R+S+ had the highest belief score (2.06 ± 0.932), followed by R+S- (1.75 ± 0.98), while R-S+ and R-S- had less faith in their Guardian Angel (1.27 ± 1.16 resp. 1.14 ± 1.04). The highest belief scores were found in widowed individuals (2.33 ± 0.58) and those > 70 years of age (2.25 ± 0.96), while the lowest score was found in subjects < 30 years (1.13 ± 0.83). Women and men did not significantly differ in their belief scores.

### Analyses of variance

Next we tested the impact of several variables on the SpREUK P sub-scales, such as age, sex, marital status, educational level, religious affiliation, SpR attitude, disease and duration of disease. Using the method of univariate analyses of variance we identified several sources of variability (Table 3):

**Table 3:** Univariate variance analyses

	**variables**	Levene's test *	**F-value**	**p-value **
**(1) conventional religious practice**	SpR attitude age	0.000	45.165 1.350	**0.000** n.s.
	religious affiliation educational level	0.007	23.192 1.396	**0.000** n.s.
				
(2) existentialistic practice	SpR attitude age	0.000	15.064 0.921	**0.000** n.s.
	religious affiliation educational level	0.028	3.557 2.768	0.030 0.040
	gender marital status gender * marital status	n.s.	4.390 2.276 2.325	**0.037** **0.029** 0.056
				
**(3) unconventional spiritual practice**	SpR attitude age	0.000	29.344 0.982	**0.000** n.s.
	religious affiliation educational level	0.003	0.417 4.429	n.s. **0.005**
	disease duration of disease	0.001	2.555 1.228	0.042 n.s.
				
**(4) humanistic practice**	disease duration of disease disease * duration of disease	0.053	2.047 1.711 3.188	0.091 n.s. **0.002**
				
**(5) nature-oriented practice**	SpR attitude age	0.000	4.058 0.302	**0.008** n.s.
	disease duration of disease	n.s.	2.484 1.088	**0.046** n.s.

In this table, only significant results were given.

*Levene's test for equality of variances was significant and thus the level of significance for the variance analyses should be p < 0.01

• **SpR attitude **is an important covariate for four of the distinct forms of practice (CRP, NoP, ExP and USP), but not for the HuP.

• **Religious affiliation **is an important covariate for CRP and ExP, but not for USP.

• **Educational level **is a covariate for USP and ExP.

• **Gender **and**Marital status **are covariates only for ExP.

• **Age **is not a relevant covariate for any of the five forms of SpR practice.

• **Disease **itself has an impact on NoP (and a minor impact on HuP and USP), while the **duration of disease **has no impact on the forms of SpR practice. However, disease and its duration are of relevance for an engagement in HuP.

### Correlation between engagement in the different forms of SpR practice and SpR attitude

Bivariate correlation between the five forms of a SpR practice revealed a strong correlation between NoP and ExP, and between ExP and USP, while CRP did not correlate with NoP or HuP, regardless of their SpR attitude (Table 4). For individuals with a spiritual attitude (R+S+ and R-S+), their ExP is strongly associated with USP and NoP (Table 4), while the ExP of R+S- individuals correlates only with NoP, but not with USP. Moreover, for those with a spiritual attitude (R+S+ and R-S+), their USP correlated well with CRP. However, the ExP of in R-S- individuals significantly correlated with a CRP.

**Table 4:** Pearson correlation between SpREUK-P sub-scales with respect to SpR attitude

	**CRP**	ExP	**USP**	**HuP**	**NoP**
**all individuals**					
conventional religious practice	1.000	.230 **	.371 **	.027	.066
existentialistic practice		1.000	.490 **	.316 **	.512 **
unconventional spiritual practice			1.000	.199 **	.302 **
humanistic practice				1.000	.220 **
nature-oriented practice					1.000

**R+S+ individuals**					
conventional religious practice	1.000	.019	.324 **	-.047	-.030
existentialistic practice		1.000	.429 **	.278 **	.505 **
unconventional spiritual practice			1.000	.216**	.307 **
humanistic practice				1.000	.284 **
nature-oriented practice					1.000

**R+S- individuals**					
conventional religious practice	1.000	.179	.255 *	.119	.000
existentialistic practice		1.000	.165	.276 *	.450 **
unconventional spiritual practice			1.000	.070	.090
humanistic practice				1.000	.'157
nature-oriented practice					1.000

**R-S+ individuals**					
conventional religious practice	1.000	.056	.400 **	-.167	.166
existentialistic practice		1.000	.465 **	.311 **	.581 **
unconventional spiritual practice			1.000	.055	.216
humanistic practice				1.000	.329 *
nature-oriented practice					1.000

**R-S- individuals**					
conventional religious practice	1.000	.410 **	.227	.083	-.030
existentialistic practice		1.000	.268 *	.313 *	.396 **
unconventional spiritual practice			1.000	.164	.153
humanistic practice				1.000	.015
nature-oriented practice					1.000

Bivariate correlations are statistically significant with * p < 0.05 and ** p < 0.01 (2-tailed significance)

As shown in Table 5, Pearson's correlation between the SpR practice and the distinct SpR measures of the SpREUK manual revealed strong correlations:

**Table 5:** Pearson correlation between SpREUK sub-scales and SpR practice

	**Search Meaning**	**Message Disease**	**Trust Guidance**	**Support External**	**Support Internal**
**SpREUK-P engagement scores**					
conventional religious practice	.381 **	.093	.659 **	.472 **	.367**
existentialistic practice	.510 **	.465 **	.361 **	.487 **	.433 **
unconventional spiritual practice	.551 **	.385 **	.330 **	.560 **	.479 **
humanistic practice	.243 **	.154 **	.014	.130 *	.112
nature-oriented practice	.322 **	.284 **	.190 **	.314 **	.367 **
(Guardian Angel)	.327 **	.271 **	.421 **	.179 *	.361 **

Bivariate correlations are statistically significant with * p < 0.05 and ** p < 0.01 (2-tailed significance)

• **CRP **correlates with "Trust in external guidance" and "Support in relations with the External life through SpR", but not with "Positive interpretation of disease".

• **ExP **correlates well with "Search for meaningful support", "Support in relations with the External life through SpR" and "Positive interpretation of disease".

• **USP **correlates well with "Support in relations with the External life through SpR" and "Search for meaningful support".

• **HuP **did not correlate at all with "Trust in external guidance" or "Support in relations with the Internal life through SpR", but marginally with both "Support through SpR" sub-scales.

• With respect to **NoP**, we found moderate correlations with all five SpREUK subscales.

The item "Belief in (my) Guardian Angel" which was used only as a preliminary marker item correlated moderately with ExP (r = 0.301) and NoP (r = 0.290), and with the SpREUK scales "Search for meaningful support" (r = 0.327) and "Support of the Internality through SpR" (r = 0.361), but somewhat higher with "Trust in external Guidance" (r = 0.421). Surprisingly, also individuals without any religious affiliations reported that they often/frequently believed in their Guardian Angel (36%, resp. 46% of R-S+ and 27% of R-S-), in contrast to 62% of the Christians (69% R+S+ and 62% of R+S-), and 86% of those with non-Christian affiliations.

## Discussion

We have confirmed the structure and consistency of the previously described SpREUK-P manual [26,27], which is an integral part of the SpREUK construct [24-28], and improved the quality of the current construct by adding several new items. Apart from conventional religious and unconventional spiritual practices, three other distinct forms of engagement were of relevance to the patients with life-threatening diseases, i.e. existentialistic practice, humanistic practice, and nature/environment-oriented practice. The latter three topics are obviously more philosophical forms of SpR.

When confronted with a life-threatening disease, more existentialistic or self-centered issues become relevant to the patients. Existentialistic philosophers (such as Søren Kierkegaard and Jean-Paul Sartre) emphasized the universal struggle to find meaning in life, to live by moral standards, and to come to an understanding of suffering and death [31,32]. To them, life might be without inherent meaning (existential atheists) or it might be without a meaning we can understand (existential theists). Consequently, since man is ultimately alone, one is free to pick and choose one's own values, and to create one's own suitable religious patchwork. In accordance with these views we found strong correlations between ExP and the SpREUK scales "Search for meaningful support" and "Positive interpretation of disease". Moreover, the ExP engagement score was much higher compared to the more formalized practices, i.e. CRP and USP. The highest engagement scores were found for HuP and NoP. One could speculate that these forms reflect a higher level of "insight", but it is also true that less effort is needed to turn to others (HuP) and nature (NoP) than to reflect on yourself (ExP); and their social desirability is much higher. One the other hand, within recent decades, ecological issues and people's appreciation of nature ("earth connection") have gained much attention, and thus higher agreement levels are not surprising. Engagement in an ExP is significantly dependent on the SpR attitude (low engagement level were found for R-S- individuals and those without any religious affiliation); gender and marital status are also relevant variables. One may speculate that divorced individuals, who have the highest ExP engagement level, reflect more on themselves because of the process of divorce ("liberation", "self-realization").

A more self-centered attitude is also measured in a scale of Holland's Spiritual Beliefs Inventory (SBI-15R-D) [19,33]: The underlying attitude of a social support through a religious faith community can be described as "What will others do for me?" In our HuP and NoP scales, the question is "What can I do for others, for nature and environment?" These contrasting views are highly affected by the state of "insight" an individual has developed.

The items of the scale HuP are related to the views of Secular Humanism [34,35] and the Philosophical resp. Christian Humanism [36]. Secular Humanism is an atheistic and naturalistic philosophy promoting humanity as the measure of all things, and roots in the rationalism of the 18th Century and the free-thought movement of the 19th Century. Secular Humanists reject the concept of a personal creator God, and regard man as fully responsible for the future of the world, its political systems, its ecology, etc. [34,35]. Thus, it is not surprising that the scale HuP neither correlates to CRP nor to the SpREUK scale "Trust in External Guidance". In fact, it correlates somewhat better with ExP and the SpREUK scale "Search for meaningful support". Consequently, the lowest HuP engagement levels were found for individuals lacking a spiritual attitude (R+S- and R-S-). Low engagement levels were also found with respect to higher age, and in patients with cancer and MS. The impact of disease and its duration on HuP remains to be explained in further studies.

To our surprise, one of the most accepted topics defining conventional religious practice, "going to church" resp. "service attendance", had a low engagement score among the German individuals tested. The same is true for the participation in religious events, while praying seems to be much more attractive. The items from the USP scale had low engagement scores too, even meditation, which is highly valued in several other questionnaires.

The presumption that both scales do not measure what they are intended to do can be rejected both from a statistical but also from a contextual point of view, because individuals with a Christian affiliation had significantly higher engagement scores for CRP than those with other religious affiliations or none, while individuals with non-Christian affiliations had the highest scores for USP. Moreover, a religious attitude (R+S+ and R+S-) was associated with significantly higher mean levels for CRP than subjects with a spiritual attitude (R-S+ and R-S+), while in contrast a spiritual attitude (R+S+ and R-S+) was associated with higher levels for USP. In this context it is worth mentioning that an R-S- attitude was associated with the lowest engagement scores for all five forms of a SpR practice. Variance analyses confirmed that SpR attitude and religious affiliation are the main relevant covariate for CRP, while for USP, the SpR attitude and the educational level are of significance, but not religious affiliation.

The level of engagement in CRP also depends on the professional background of the tested subjects. We found that the engagement score was very high in attendants of a Christian Caritas meeting (mainly priests, chaplains, Christian social workers etc.) and in composers of Contemporary Christian Songs (mean values 79.9 ± 20.3 resp. 77.0 ± 13.1); high scores were found also in attendants of a meeting on "Spirituality and Health" (56.9 ± 33.3), while the lowest CRP score was found in hospital staff (32.7 ± 20.0). The engagement score of catholic nurses caring for out-patients (45.7 ± 25.6) was similar to the overall mean level (47.1 ± 28.8). Details of this investigation will be presented elsewhere.

Using the German version of Holland's Spiritual Beliefs Inventory (SBI-15R-D), Albani *et al*. found that higher religiosity is observed for women, older people, people with lower education, former West Germans vs. former East Germans, and people stating a religious affiliation [33]. These findings are only in part congruent with ours: The higher CRP engagement score in women was not statistically significant, while lower age and lower educational level were associated with significantly lower CRP engagement scores. However, we can confirm higher scores for patients with a Christian affiliation and a religious attitude (R+S+ and R+S-). Using the SpREUK 1.1 inventory, we have found that women with cancer have significantly higher scores for "Search for meaningful support", "Interpretation of disease" and "Support in relations with the external life through SpR", but not for "Trust in external Guidance"[24] Similar, cancer patients with a lower educational level had significantly lower scores for "Search for meaningful support" and "Interpretation of disease", though again not for "Trust in external Guidance" [24]. Thus, it is obvious that the condensed 10-item scale of the shortened SB-15-R [19], which measures mainly religious beliefs and convictions dealing with the support through God and faith, represents only a distinct aspect of religiosity.

In fact, "religiosity" is already multidimensional construct. Batson *et al*. described a three-dimensional model of religiosity: Means or external, End or internal, and Quest [14, 37]. Intrinsic religiosity identifies religion as an end in itself. Strong personal convictions, beliefs and values are what matter, while the social aspects of religion are not that important. In contrast, the motifs of extrinsic religiosity are based on social or external values and beliefs; religion is used to gain social standing and endorsement. The Quest orientation is founded on a willingness to question complex ideas. The persons are open to the exploration of existential questions and they are open for new information and doubts. Thus, as we have to assume a complex interconnection of various existing views, attitudes and concepts, an oversimplification ("two scales are enough") of SpR concerns in QoL research is not appropriate.

## Conclusion

Our scales are in congruence with external factors influencing the distinct forms and frequency of a patients SpR engagement. The SpREUK questionnaire with its SpREUK-P manual thus could be of value in measuring SpR attitudes and engagement of patients coping with life-threatening illness, and in the measurement of distinct aspects of QoL.

An advantage of our instruments is the clear-cut differentiation between convictions and attitudes on the one hand, and the expression of these attitudes in concrete engagement on the other. A second advantage is the differentiation of five distinct forms of spiritual, religious and philosophical practice. Finally the fact that the validation was performed in a sample with at least two different types of life-changing diseases (cancer and MS, and other chronic diseases) and a healthy control group is advantageous for interpreting the results.

In future studies we will emphasize the correlation our scales with conventional QoL instruments. Nevertheless, evaluation of the SpREUK-P questionnaire indicates that it is a reliable, valid measure of distinct topics of SpR practices. The focus of a larger study is to enroll patients from the highly secular Eastern Europe, and to run longitudinal studies with cancer, multiple sclerosis patients, but also cardiac failure and spinal cord damage.

The conventional SpREUK (Version 1.1) and its SpREUK-P manual (Version 1.1) are currently available in English and German.

## Authors' contributions

AB conceived the study, designed and developed the questionnaire, performed statistical analysis and drafted the manuscript. TO participated to conceive and design the study assisted in statistical analysis and helped to draft the manuscript. PFM participated in the design and development of the questionnaire. All authors read and approved the final manuscript.
